# Zolbetuximab or Immunotherapy as the Initial Targeted Therapy in CLDN18.2-Positive, HER2-Negative Advanced Gastric Cancer: Weighing the Options

**DOI:** 10.3390/curroncol32110648

**Published:** 2025-11-20

**Authors:** Jacob C. Easaw, Howard J. Lim, Hatim Karachiwala, Sharlene Gill, Xiaofu Zhu, Justin Bateman

**Affiliations:** 1 Faculty of Medicine & Dentistry, Department of Oncology, University of Alberta, Edmonton, AB T6G 2R3, Canada; xiaofu.zhu@albertahealthservices.ca; 2 Division of Medical Oncology, Faculty of Medicine, University of British Columbia and Systemic Therapy BC Cancer, Vancouver, BC V5Z 4E6, Canada; hlim@bccancer.bc.ca (H.J.L.); sgill@bccancer.bc.ca (S.G.); 3 Division of Medical Oncology, Arthur J.E. Child Comprehensive Cancer Center, Calgary, AB T2N 5G2, Canada; hatim.karachiwala@albertahealthservices.ca; 4 Faculty of Medicine & Dentistry, Laboratory Medicine & Pathology Department, University of Alberta, Edmonton, AB T6G 1C9, Canada; justin.bateman@albertaprecisionlabs.ca

**Keywords:** biomarkers, claudin-18.2, gastric cancer, immune checkpoint inhibitors, PD-L1 CPS, zolbetuximab

## Abstract

Advanced gastric/gastroesophageal junction (G/GEJ) adenocarcinoma remains a common and deadly form of cancer. Advances in G/GEJ cancer treatment have improved survival outcomes with the claudin-18.2 (CLDN18.2)-targeted agent, zolbetuximab, and immune checkpoint inhibitors (ICIs) targeting the PD-1 receptor. This article offers an evidence-informed opinion on considerations when selecting between these first-line treatments for G/GEJ adenocarcinoma in patients with HER2-negative disease that expresses CLDN18.2 and/or PD-L1, including the reliability of biomarker scoring and interpretation, overall survival (OS) rates, toxicity profiles, and logistical practicalities. Evidence from Phase III trials for zolbetuximab and ICIs suggest similar OS benefits of 14–18 months compared to chemotherapy alone, but there appears to be a gradient of benefit for ICIs with increasing PD-L1 combined positive score (CPS). There is high inter-observer variability in CPS scoring, particularly at lower thresholds. Zolbetuximab is associated with high rates of nausea and vomiting during the initial infusion, whereas ICIs are associated with risk of later-onset immune-related toxicities that can be fatal in rare cases. In considering the available evidence, our opinion is that zolbetuximab is a reasonable option for initial targeted treatment in HER2-/CLDN18.2-positive advanced G/GEJ when PD-L1 CPS score is <10 based on the reliability of biomarker testing, comparable OS, and avoidance of potentially irreversible ICI-induced immune toxicity.

## Simple Summary

Targeted treatments for advanced gastric cancer have been shown to improve survival when added to conventional chemotherapy. A claudin18.2-targeted drug, zolbetuximab, and two immune checkpoint inhibitors, nivolumab and pembrolizumab, are now approved as initial therapies for advanced gastric cancer. Since these drugs offer similar survival benefits, choice of initial therapy hinges on their side-effect profiles, patient factors, tumor characteristics such as biomarker expression, and logistical practicalities. Zolbetuximab has high rates of nausea and vomiting during the first infusion, but rates are lower with subsequent infusions and can be managed with anti-nausea medications. Nivolumab and pembrolizumab carry a risk of potentially serious immune side effects that can occur weeks after starting treatment. All three therapies work best against tumors that express specific biomarkers or targets. This makes the reliability of biomarker testing and interpretation an important factor when choosing initial therapy for advanced gastric cancer.

## 1. Introduction

Gastric and gastroesophageal junction (G/GEJ) cancer is the fifth most common form of cancer, with more than one million new cases diagnosed worldwide in 2020 [1]. It is the fourth most deadly cancer, with dismal outcomes, including five-year survival of only 5–10% in advanced disease [2]. Immune checkpoint inhibitors (ICIs) have transformed the standard of care for G/GEJ cancer. In patients with programmed cell death ligand-1 (PD-L1)-positive disease, ICIs have been shown to provide clinically meaningful improvements in overall survival (OS) in Phase III trials. This benefit has largely been driven by tumors with a PD-L1 combined positive score (CPS) ≥10 [3,4]. Recently, important strides in tumor biology have spurred the identification of new targets for treatment of G/GEJ cancer. One such target is claudin-18.2 (CLDN18.2), which is a tight junction protein that becomes exposed and targetable during malignant transformation [5,6,7]. Zolbetuximab is a first-in-class chimeric IgG1 monoclonal antibody against CLDN18.2, which is present on the surface of approximately 40% of primary or metastatic G/GEJ tumors [8]. With its recent approval in several jurisdictions, oncologists now face the challenge of choosing between zolbetuximab and ICI for first-line treatment of HER2-/CLDN18.2+ G/GEJ adenocarcinomas in dual biomarker-positive tumors that also co-express PD-L1 with CPS ≥1. In the absence of direct head-to-head comparative clinical trial data, the goal of this article is to provide an evidence-informed expert opinion of the benefits and caveats of zolbetuximab vs. ICIs based on their respective Phase III trials to help oncologists personalize front-line treatment for patients who are potentially eligible for both treatments. The authors identified factors that influence the selection of initial therapy in G/GEJ adenocarcinoma, and targeted literature searches were subsequently conducted to support each topic. Relevant articles were reviewed by the authors for inclusion in the manuscript.

## 2. Efficacy and Safety Data from the Respective Phase III Studies

The clinical effectiveness of ICIs and zolbetuximab for the treatment of advanced G/GEJ adenocarcinoma is supported by independent Phase III registration studies for each agent: CheckMate-649 for nivolumab [3,9], KEYNOTE-859 for pembrolizumab [4], and SPOTLIGHT [10] and GLOW [11] for zolbetuximab. These studies differ with respect to design, geographic representation, chemotherapy backbones, enrolled patient populations, and baseline disease characteristics (see Supplemental Table S1). In the absence of direct head-to-head trials, comparisons are descriptive rather than inferential.

### 2.1. Immune Checkpoint Inhibitors—Efficacy and Toxicity

CheckMate-649 was a Phase III randomized, open-label trial in adults with first-line, unresectable, HER2-, advanced or metastatic G/GEJ adenocarcinomas, regardless of their PD-L1 CPS score [3]. A total of 1581 patients were randomized to receive either (i) nivolumab + chemotherapy (XELOX Q3W or FOLFOX Q2W), (ii) nivolumab + ipilimumab, or (iii) chemotherapy alone. The trial showed that the addition of nivolumab to chemotherapy resulted in improved OS (hazard ratio [HR] 0.79 at 12.1 months) and progression-free survival (PFS) in the overall population (Table 1), with the greatest benefit observed in patients with higher CPS scores [3,9].

The Phase III KEYNOTE-859 trial randomized 1579 patients with treatment-naïve, HER2-, advanced G/GEJ adenocarcinoma to receive either pembrolizumab + chemotherapy (FP or CAPOX) or chemotherapy alone [4]. Outcomes were significantly improved with the addition of pembrolizumab to chemotherapy (Table 1), including a significant OS benefit (HR 0.78 after a median of 31 months follow-up). Again, the magnitude of benefit increased with higher PD-L1 expression levels.

**Figure 1:**
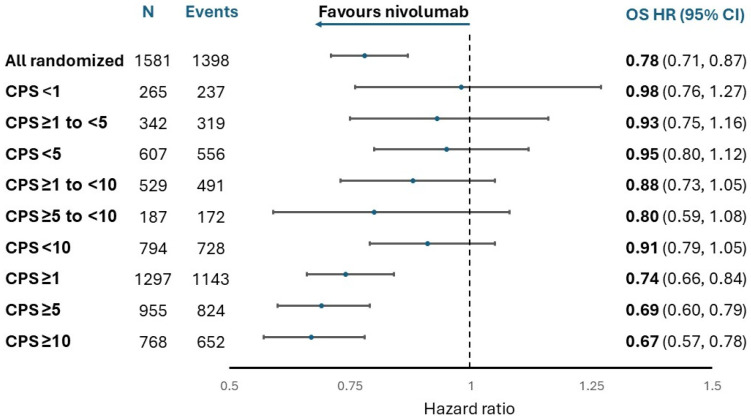
Forest plot of nivolumab’s effect on overall survival by PD-L1 CPS status in all randomized patients (4-year follow-up). Adapted from: Sponsor briefing document provided by BMS to the FDA’s Oncologic Drugs Advisory Committee (ODAC) meeting on 26 September 2024. Available at https://www.fda.gov/advisory-committees/advisory-committee-calendar/september-26-2024-meeting-oncologic-drugs-advisory-committee-meeting-announcement-09262024 (accessed on 30 April 2025).

**Figure 2:**
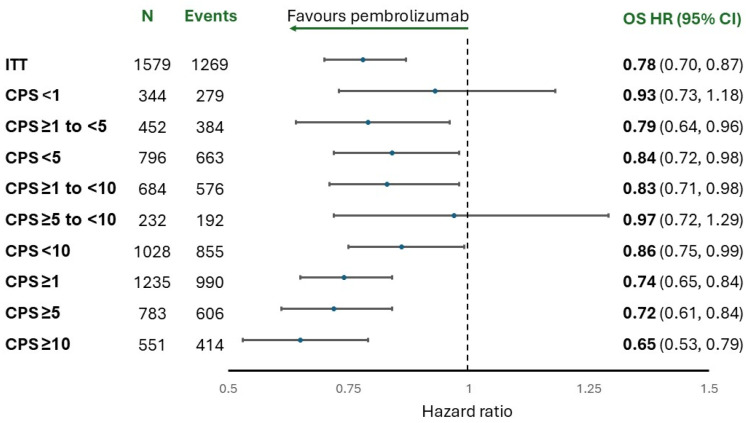
Forest plot of nivolumab’s effect on overall survival by PD-L1 CPS status in all randomized patients. Adapted from: Sponsor briefing document provided by Merck to the FDA’s Oncologic Drugs Advisory Committee (ODAC) meeting on 26 September 2024. Available at https://www.fda.gov/advisory-committees/advisory-committee-calendar/september-26-2024-meeting-oncologic-drugs-advisory-committee-meeting-announcement-09262024 (accessed on 30 April 2025).

Collectively, these trials show consistent, clinically relevant benefit from PD-1 blockade, particularly in tumors with CPS ≥10 and no meaningful benefit when CPS is <1 [4,9]. The benefit is less clear for tumors with CPS 1–9. To address this, the U.S. Food and Drug Administration (FDA) evaluated OS outcomes by different PD-L1 CPS thresholds using the data for nivolumab (Figure 1) and pembrolizumab (Figure 2). Overall, an OS benefit was not shown at lower thresholds (CPS < 1, CPS < 5, and CPS < 10) or within the “grey zones” of CPS 1 to <5 and CPS 5 to <10. An important limitation of these analyses is that the cohorts for these PD-L1 CPS scores are small (<100 for nivolumab), and the confidence intervals are wide. Nonetheless, the findings suggest that OS with ICIs occurs along a gradient, with no benefit when CPS is <1 and questionable benefit when CPS is <10. This makes the reliability of PD-L1 biomarker testing a critically important consideration when selecting initial therapy for G/GEJ adenocarcinoma. A similar analysis was conducted by the European Medicines Agency, with comparable findings.

ICI therapies have a favorable overall tolerability profile; however, a concern with these agents is the longer-term risk of serious immune-related adverse events (irAEs) such as hypothyroidism, colitis, myocarditis, immune nephritis, and cutaneous toxicities [12]. Although infrequent, irAEs can be serious and even fatal, they are difficult to predict, and they often have delayed onset (ranging from a few days to several months, with a median time to onset of 40 days) [12,13,14]. In the Phase III trials of ICI therapies in G/GEJ adenocarcinoma, treatment-related adverse events that had a potentially immunologic etiology were most commonly gastrointestinal, hepatic, cutaneous, and thyroid-related, although the type and incidence varied by ICI therapy (Table 2) [4,9]. In the KEYNOTE-859 study, there were two grade 5 pneumonitis events (one in the pembrolizumab arm and one in the placebo arm) [4]. Interventions for irAEs consist of high-dose systemic corticosteroids, immunomodulators (e.g., infliximab), and ICI discontinuation if irAEs are grade ≥2 [14]. Although many irAEs can be controlled or reversed after 4–8 weeks, they can have a prolonged course and require long-term treatment. In a meta-analysis of irAEs associated with ICI therapies in G/GEJ, the overall incidence of irAEs was 16% (all grades); 3% were severe, and 1% were fatal, mostly due to colitis and interstitial lung disease [13].

### 2.2. Zolbetuximab—Efficacy and Toxicity

The efficacy and safety of zolbetuximab were investigated in two Phase III, randomized, double-blind, placebo-controlled trials (SPOTLIGHT and GLOW) in adults with previously untreated HER2-/CLDN18.2+, locally advanced unresectable or metastatic G/GEJ adenocarcinoma [15,16]. These trials were identically designed, except for the comparator chemotherapy arms—modified FOLFOX (mFOLFOX) Q2W in SPOTLIGHT or CAPOX Q3W in GLOW. In a pooled analysis of both trials, median OS (HR 0.77 at 32.7 months) and PFS were significantly improved (see Table 1 for individual trial data) [17]. Although the observed OS was ~4 months longer in SPOTLIGHT than in GLOW (18.2 vs. 14.3 months), the hazard ratios were similar (0.78 and 0.77), owing to the longer OS in the control arm (15.6 vs. 12.2 months) [15,16]. It has been suggested that differences in the patient populations enrolled in the two trials might account for the better survival in SPOTLIGHT—notably, a greater contribution of patients from Japan and Korea, where G/GEJ prognosis is generally better, whereas GLOW enrolled more patients from mainland China [18]. Recently, health authorities have approved Q2W or Q3W dosing schedules for zolbetuximab, allowing it to be dosed synchronously with either chemotherapy backbone (i.e., mFOLFOX or CAPOX) [19].

Nausea and vomiting (N/V) were the most common adverse events in both trials (nausea: 76% all grades with zolbetuximab vs. 56.2% with placebo; vomiting: 66.8% all grades with zolbetuximab vs. 34.2% with placebo) [20]. Importantly, there was a high rate of nausea and vomiting events that were grade 3 or higher (nausea: 12.6% with zolbetuximab vs. 4.7% with placebo; vomiting: 14.3% with zolbetuximab vs. 4.9% with placebo). Moreover, N/V events usually occurred within <1 h of the first infusion. The occurrence of nausea and vomiting decreased by 69% and 65%, respectively, from the first to the second infusion, and both events were rare during all subsequent infusions [15,16,21].

It is possible that N/V rates during the first infusion were elevated because antiemetic and infusion schedules were not optimal. Notably, the use of dexamethasone was discouraged [15,16]; thus, only 34–44% of patients received prophylactic steroids, which may have contributed to the high rates of N/V [21]. A post hoc analysis of the Phase III trials showed that PFS and OS were similar in the subgroup of patients that received dexamethasone compared to the overall study population, suggesting that there was no detrimental effect of corticosteroids on zolbetuximab efficacy [21]. Findings from a real-world observational trial suggest that a slower initial infusion rate in cycle 1 and aggressive use of a quadruple drug antiemetic regimen comprising a 5-HT3 receptor inhibitor, NK1 receptor inhibitor, dexamethasone, and olanzapine can substantially reduce the incidence of N/V with zolbetuximab [22]. Indeed, the incidence of vomiting during cycle 1 was reduced to 19% using this strategy, which is comparable to the rates observed in the placebo arm of the SPOTLIGHT and GLOW trials (17.5%). Although dose interruptions due to N/V were still common (62% of patients), all patients successfully completed zolbetuximab infusion in cycle 1 [22]. Zolbetuximab was well tolerated during cycle 2, with 0% of patients experiencing vomiting, compared to 26% in SPOTLIGHT and GLOW. A 2025 Delphi panel recommended a triple or quadruple antiemetic regimen prior to the first infusion and, if N/V still occurs, temporarily pausing the zolbetuximab infusion for 30–60 min and restarting the infusion at half speed; modifications to the dose are not recommended [23].

## 3. Biomarker Testing in G/GEJ Adenocarcinoma

Management of G/GEJ adenocarcinomas has evolved with the identification of predictive biomarkers. Canadian expert consensus guidelines recommend that patients with G/GEJ be tested for HER2, MMR and CPS (PD-L1)—and now, CLDN18.2 [24]—in order to personalize first-line therapy [25,26]. Now that zolbetuximab is approved and offers an alternative first-line option to ICI, it is essential to have all biomarker results upfront, and the reliability of those biomarkers is paramount.

CLDN18.2 is detected using a standard immunohistochemistry (IHC) assay [27]. In the Phase III trials for zolbetuximab, a positive result with respect to zolbetuximab treatment eligibility was defined as ≥75% of tumor cells exhibiting moderate to strong membranous staining using a validated assay such as the commercially available VENTANA CLDN18 (43-14A) RxDx IHC assay [15,16]. While this is the accepted clinical standard, other studies have used lower cutoffs to define CLDN18.2 positivity (i.e., ≥40% and ≥50%), which could potentially broaden the clinical applicability of CLDN18.2-targeted therapies [28]. A global ring study determined that CLDN18.2 can be measured with a high degree of accuracy between laboratories [29], with approximately 40% of tumors being CLDN18.2+ [8].

PD-L1 CPS is technically challenging in terms of staining and scoring, leading to low inter-pathologist reproducibility [30,31]. The most common scoring method is the CPS, which is the ratio of PD-L1 staining cells (including tumor cells and immune cells) to the number of viable tumor cells [24], with scores reported as whole numbers from 0 to 100. Different diagnostic assays are linked to different drugs (28-8 for nivolumab in CheckMate-649 [3] and 22C3 for pembrolizumab in KEYNOTE-859 [4]), and the two assays are not necessarily interchangeable without validation [25,32]. Therefore, institutions that use both ICIs need both assays—or must validate one against the other—adding cost and complexity. Moreover, there is no established cutoff for PD-L1 CPS positivity, and different cutoffs have been used in clinical trials [3,4], making cross-trial comparisons difficult. Furthermore, there is variability in how different laboratories report PD-L1 CPS scores. For example, in Vancouver, British Columbia, where PD-L1 CPS testing is centralized, results are reported as <1, 1–9, or ≥10. In Ontario and Quebec, there is no standard CPS cutoff reported, with some institutions reporting discrete values including up to one decimal (e.g., 7.4). Another limitation is that inter-observer agreement in CPS interpretation is poor, particularly when CPS is <10 [30,31]. One study reported only 31% overall agreement at the <1 vs. ≥1 threshold when comparing the results of 14 pathologists, improving to only 68% at the CPS 10 threshold [30]. Similarly, a study of 12 pathologists who assessed 100 biopsy samples with both 22C3 and 28-8 assays reported only 45–55% baseline concordance, rising to 56–57% after dedicated training [31]. These data underscore that CPS, particularly at cutoffs <10, is not a highly reproducible biomarker in real-world practice [30,31].

**Figure 3:**
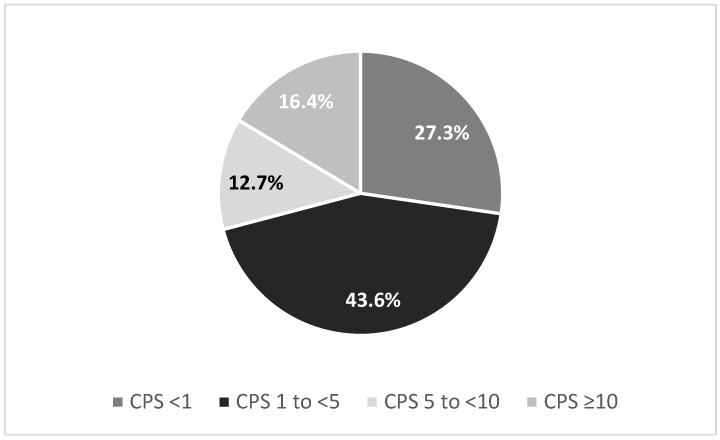
Distribution of PD-L1 CPS scores by different cutoffs in G/GEJ tumor resections [33].

Roughly one-third of G/GEJ tumors have CPS ≥ 5 (Figure 3) [33]. In the SPOTLIGHT and GLOW trials, only 17% of CLDN18.2+ tumors had CPS ≥ 5 [8], suggesting that the number of dual biomarker expression-positive patients where the physician will need to choose between ICI and zolbetuximab is likely to be small. However, given the reliability of CLDN18.2 testing and the similar efficacy benefit seen with zolbetuximab and ICIs, zolbetuximab is a reasonable option for HER2-/CLDN18.2+ tumors that fall into the “grey zone” of CPS 1-–9, where test reliability is lowest and the clinical advantage of ICIs is modest. Importantly, 82% of patients in SPOTLIGHT and GLOW had CPS < 5 [8] and experienced a survival advantage from zolbetuximab, supporting its use when CPS does not clearly justify immunotherapy.

## 4. Considerations Related to Clinical Practicalities

When considering patient characteristics that favor treatment with zolbetuximab or ICI therapies, primary tumor site does not appear to be a key factor based on evidence from clinical trials. Prespecified subgroup analyses of the CheckMate-649, SPOTLIGHT, and GLOW studies showed no statistically significant OS benefit from nivolumab or zolbetuximab in the subset of patients with GEJ adenocarcinoma [9,15,16]. However, these constituted small subgroups, and the confidence intervals are wide; thus, the results can only be hypothesis-generating. In contrast, the OS benefit of pembrolizumab was preserved in the subgroup of patients with GEJ tumors [4]. The lower observed responsiveness of GEJ compared to gastric tumors is not surprising, since there are differences in the tumor immune microenvironment between these tumors that can affect treatment responsiveness [34]. Furthermore, patients with GEJ tend to have worse disease-specific survival and higher recurrence rates compared to gastric cancer [35]. Generally, in Canadian clinical practice, gastric and GEJ adenocarcinomas are treated the same, and it is unlikely that findings from a subgroup analysis will change this approach.

Logistical realities may also factor into the choice between zolbetuximab and ICIs, particularly in situations where benefits and risks might otherwise be similar. In this respect, when following recommended administration schedules, the first zolbetuximab infusion takes a minimum of ~3.5 h, on average (range: 3.33–4.5 h) [21], not considering infusion delays and interruptions to mitigate against N/V. Real-world experience with the implementation of antiemetic and N/V control procedures [23] is eagerly awaited to better understand the practical realities of zolbetuximab infusion. Of note, Health Canada and other health authorities have approved Q2W or Q3W dosing schedules for zolbetuximab, allowing it to be dosed synchronously with either chemotherapy backbone (mFOLFOX or CAPOX) [19].

In favor of ICIs is the fact that medical oncologists have more experience with this class of therapy across multiple indications and are, therefore, more comfortable with management of these treatments. However, the impressive responses observed in some tumors, such as metastatic melanoma [36], have not translated to G/GEJ [4,9]. Indeed, the opportunity for long-lasting disease remission in melanoma (i.e., the chance of “getting on the tail of the OS curve”) is appealing, but it should be noted that in G/GEJ, the best chances for long-term survival with ICI therapies is in patients with PD-L1 CPS ≥ 10 [4,9]. Moreover, disease control can be durable with zolbetuximab (i.e., median OS of 18.23 months in SPOTLIGHT) and, thus, offers patients comparable potential for long-lasting disease control [10,15,16,20].

## 5. Future Directions

The treatment landscape in G/GEJ adenocarcinoma is evolving, and clinicians can now offer patients a choice of targeted therapies that have demonstrated survival benefits. This is an area of active research interest, and there are other novel and emerging therapies in various stages of development for G/GEJ, including CAR-T therapies, FGFR2b bispecific antibodies, antibody–drug conjugates, other novel monoclonal antibodies, and vaccines [37,38]. The impact of treatment sequencing remains a topic of debate that is not yet well supported by evidence and may be impacted by drug reimbursement policies. Emerging evidence suggests that CLDN18.2 expression is dynamic in up to one-quarter of G/GEJ tumors during chemo-immunotherapy, with some tumors exhibiting loss of CLDN18.2 and others gain of CLDN18.2 positivity [39]. Moreover, tumors that converted to CLDN18.2+ exhibited a unique tumor microenvironment dominated by stromal and fibrotic remodeling [39]. These observations suggest potential benefits of serial CLDN18.2 monitoring over time and support the utility of combination anti-CLDN18.2 plus ICI therapies in some biomarker-defined subgroups. The role of combination zolbetuximab + ICI + chemotherapy is currently being investigated in a Phase II trial (ILUSTRO) [40]. The results of ongoing studies are eagerly anticipated, as they hold promise to further improve the dismal outcomes in G/GEJ.

## 6. Conclusions

**Figure 4:**
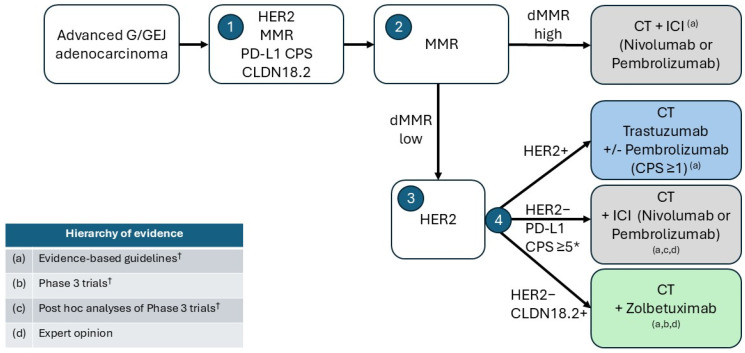
Proposed stepwise decision algorithm for choice of first-line treatment of advanced G/GEJ adenocarcinoma based on evidence hierarchy and according to biomarkers. 1. Concurrent biomarker testing for four key G/GEJ biomarkers [25]; 2. Consider MMR deficiency; if high, ICI may be added to CT [26,41] 3. if dMMR-low, consider HER2 status; 4. If HER2-positive, consider adding trastuzumab +/− pembrolizumab to CT [26,41] (depending on local regulatory approval and reimbursement), and if HER2-negative, consider PD-L1 CPS score and CLDN18.2 status [4,9,30,31,41] [and expert opinion]. * Evidence suggests that the greatest efficacy for ICIs is achieved when PD-L1 CPS score is ≥10. ^†^ See text for references. CLDN18.2, claudin-18.2; CPS, combined positive score; CT, chemotherapy; d/MMR, deficient/mismatch repair; HER2, human epidermal growth factor receptor 2; ICI, immune checkpoint inhibitor; G/GEJ, gastric/gastroesophageal; PD-L1, programmed cell death ligand.

Key considerations when selecting between zolbetuximab and ICIs as initial therapy for advanced G/GEJ include the availability of a reliable predictive biomarker, evidence supporting OS benefits, and safety and toxicity profiles. Although there is no head-to-head comparison between the available targeted therapies for G/GEJ cancer and notwithstanding the heterogeneity between different trial populations, some general inferences can be drawn. Considering the body of available evidence, we propose a stepwise treatment decision pathway for choice of first-line treatment of advanced G/GEJ adenocarcinoma according to four key biomarkers (Figure 4). Treatment selection for deficient MMR-high and HER2+ G/GEJ adenocarcinoma is well supported by evidence-based guidelines [26,41]. For HER2- tumors, there remain evidence gaps to inform optimal first-line treatment selection. Our expert opinion is that zolbetuximab is a reasonable option for patients with HER2-/CLDN18.2+ G/GEJ tumors when the PD-L1 CPS score is <1, whereas ICI therapies have the strongest benefit when the PD-L1 CPS score is ≥10. For patients with low CPS scores between 1 and <5, zolbetuximab could be selected for initial therapy, given the low reliability of CPS interpretation in this range, coupled with the less compelling OS outcomes with ICI therapies. The majority of patients with G/GEJ who are candidates for zolbetuximab based on HER2-/CLDN18.2+ tumors have PD-L1 CPS scores <5, where ICI therapy has marginal benefit. For intermediate CPS scores of 5 to <10, there is support for an OS benefit of ICIs; however, the reliability of CPS scoring and interpretation in this dual biomarker expression “grey zone” is a very real challenge.

The benefits and risks of any treatment need to be discussed with patients, since there can be wide variability in an individual’s risk tolerance. N/V associated with zolbetuximab usually occurs during the first few infusions and can be managed with judicious use of antiemetic regimens and infusion rate adjustments. Conversely, the irAEs seen with ICIs develop over time and can be serious and potentially life-threatening. Notably, the risk of serious irAEs, although rare, may take on greater importance when the benefits of ICI therapies are less certain (i.e., when PD-L1 CPS scores are <10).

Ultimately, treatment must be individualized, taking into account tumor biology, balanced against toxicity and aligned with each patient’s goals and preferences after an open discussion of the expected benefits and risks of appropriate treatment options.

## Figures and Tables

**Table 1:** Overall and progression-free survival outcomes for drug vs. placebo in Phase III trials for zolbetuximab, nivolumab, and pembrolizumab.

	CheckMate-649 [9]	KEYNOTE-859 [4]	SPOTLIGHT [10]	GLOW [11]
Median follow-up for OS, months	47.4	31.0	33.28	26.1
Median OS, all patients, months, HR (95% CI)	13.7 vs. 11.6,	12.9 vs. 11.5,	18.23 vs. 15.57,	14.3 vs. 12.2,
	0.79 (0.71, 0.88)	0.78 (0.70, 0.87)	0.784 (0.644, 0.954)	0.77 (0.62, 0.95)
Median OS, patients with PD-L1 CPS <1, months, HR (95% CI)	13.1 vs. 12.5,	NR	--	--
	0.95 (0.74, 1.24)	0.92 (0.73, 1.17)		
Median OS, patients with PD-L1 CPS ≥1, months, HR (95% CI)	13.8 vs. 11.3,	13.0 vs. 11.4,	--	--
	0.75 (0.66, 0.84)	0.74 (0.65, 0.84)		
Median OS, patients with PD-L1 ≥10, months, HR (95% CI)	15.0 vs. 10.9,	15.7 vs. 11.8,	--	--
	0.66 (0.57, 0.77)	0.65 (0.53, 0.79)		
Median PFS, all patients, months, HR (95% CI)	7.7 vs. 6.9,	6.9 vs. 5.6,	11.04 vs. 8.94,	8.3 vs. 6.8,
	0.79 (0.71, 0.89)	0.76 (0.67, 0.85)	0.734 (0.591, 0.910)	0.68 (0.55, 0.85)

Data from separate trials are displayed for descriptive purposes only; not meant for direct comparison. CI, confidence interval; CPS, combined positive score; HR, hazard ratio; NR, not reported; OS, overall survival; PD-L1, programmed death ligand-1; PFS, progression-free survival.

**Table 2:** Immune-related adverse events for drug vs. placebo in Phase III trials for nivolumab and pembrolizumab in G/GEJ adenocarcinoma.

	CheckMate-649 [9]		KEYNOTE-859 [4]	
	All Grades	Grade 3–4	All Grades	Grade 3–4
Gastrointestinal	34%	5%	--	--
Cutaneous	28%	4%	--	--
Hepatic	27%	4%	1%	<1%
Endocrine	14%	<1%	--	--
Renal	4%	<1%	1%	1%
Hypothyroidism	--	--	15%	<1%
Hyperthyroidism	--	--	6%	0%
Colitis	--	--	1%	2%
Pneumonitis	5%	2%	2%	1% ^1^

Data shown as reported (-- indicates data not reported). ^1^ One grade 5 event.

## Data Availability

No new data were created or analyzed in this study. Data sharing is not applicable to this article.

## Supplementary Materials

The following supporting information can be downloaded at: https://www.mdpi.com/article/10.3390/curroncol32110648/s1, Table S1: Study design and key baseline demographics and disease characteristics of the Phase III trials for ICIs and zolbetuximab in G/GEJ adenocarcinoma.

## Abbreviations

The following abbreviations are used in this manuscript:

5-HT3	5-hydroxytryptamine type 3
CAPOX	capecitabine+oxaliplatin
CAR-T	chimeric antigen receptor T-cell therapy
CI	confidence interval
CLDN	claudin
CPS	combined positive score
d/MMR	deficient/mismatch repair
FDA	Food and Drug Administration
FGFR2b	fibroblast growth factor receptor 2 isoform IIIb
FOLFOX	folinic acid+fluorouracil+oxaliplatin
FP	fluoropyrimidine
G/GEJ	gastric/gastroesophageal
HER2	human epidermal growth factor receptor-2
HR	hazard ratio
ICI	immune checkpoint inhibitor
IHC	immunohistochemistry
irAEs	immune-related adverse events
MMR	mismatch repair
N/V	nausea and vomiting
NK1	neurokinin-1
PD-L1	programmed death-ligand 1
PFS	progression-free survival
Q2W/Q3W	every 2 weeks/every 3 weeks
RxDx	companion diagnostic
