# Supplementary material for: Zolbetuximab or Immunotherapy as the Initial Targeted Therapy in CLDN18.2-Positive, HER2-Negative Advanced Gastric Cancer: Weighing the Options
Source: Curr Oncol. 2025 Nov 20;32(11):648. doi: 10.3390/curroncol32110648 (PMC12650800; doi:10.3390/curroncol32110648)
Supplement: Supplementary file 1 [file curroncol-32-00648-s001.zip › curroncol-3919689-supplementary.pdf]

Supplemental Table 1. Study design and key baseline demographics and disease characteristics of the Phase III trials for ICIs and zolbetuximab in G/GEJ adenocarcinoma

	CheckMate-649 [3]	KEYNOTE-859 [4]	SPOTLIGHT [10]	GLOW [11]
Study description				
Study design	Global, randomized, open-label, Phase III	Global, randomized, double-blind, placebo-controlled, Phase III	Global, randomized, double-blind, placebo-controlled, Phase III	
Key eligibility criteria	Previously untreated, unresectable, gastric/GEJ/ esophageal adenocarcinoma<br>No known HER2+<br>ECOG PS 0-1	Previously untreated locally advanced or metastatic G/GEJ adenocarcinoma<br>HER2-<br>ECOG PS 0-1	Previously untreated, locally advanced, unresectable or metastatic G/GEJ adenocarcinoma<br>CLDN18.2+ (moderate-to-strong staining in ≥75% of tumor cells)<br>HER2-<br>ECOG PS 0-1	
Stratification factors	PD-L1 (≥1% or <1%)<br>Region (Asia vs US/Canada vs rest of world)<br>CT (XELOX vs FOLFOX)<br>ECOG PS (0 vs 1)	PD-L1 (≥1% or <1%)<br>Region (Asia vs Europe/ Israel/North America/ Australia vs rest of world)<br>CT (CAPOX vs FP)	Region (Asia vs non-Asia)<br>No. of organs with metastases (0-2 vs ≥3)<br>Prior gastrectomy	
Chemotherapy backbone	CAPOX Q3W or FOLFOX Q2W	FP Q3W or CAPOX Q3W	mFOLFOX IV Q2W (cycles 1-4)<br>5-FU + folinic acid IV Q2W (cycles 5+)	CAPOX Q3W (cycles 1-8)<br>Capecitabine (cycles 9+)
Primary endpoint(s)	PFS (PD-L1 CPS ≥5)<br>OS (PD-L1 CPS ≥5)	OS	PFS	PFS
Key baseline characteristics				
Age, years (range)	62 (54-69)	61 (52-67)	62 (27-83)	61 (22-82)
Male sex, n (%)	68	67	62	63
Region, n (%)				
Asia	23	33	31	62
Non-Asia	77	67	69	38
Prior gastrectomy, n (%)	20	22	30	30
Primary site, n (%)				
Stomach	70	81	77	86
GEJ	17	19	23	14
EAC	13	N/A	N/A	N/A
ECOG PS, n (%)				
0	41	36	44	43
1	59	64	55	57

Data from separate trials are displayed for descriptive purposes only; not meant for direct comparison. 5-FU, 5-fluorouracil; CAPOX, capecitabine + oxaliplatin; CPS, combined positive score; CT, chemotherapy; EAC, esophageal adenocarcinoma; ECOG PS, Eastern Cooperative Oncology Group Performance Status; FOLFOX, fluorouracil + leucovorin + oxaliplatin; FP, fluorouracil + cisplatin; G/GEJ, gastric/gastroesophageal; N/A, not applicable; OS, overall survival; PD-L1, programmed death ligand-1; PFS, progression-free survival; Q2W/Q3W, every 2/3 weeks.
